# Prescription trends for combined oral contraceptives and thromboembolism incidence in Japan before and after public awareness events in 2013–2015

**DOI:** 10.1002/ijgo.70835

**Published:** 2026-01-26

**Authors:** Risa Ishida, Yusuke Sasabuchi, Kaori Koga, Shiori Itoi, Gentaro Izumi, Daisuke Shigemi, Hiroki Matsui, Yutaka Osuga, Hideo Yasunaga

**Affiliations:** ^1^ Department of Obstetrics and Gynecology, Faculty of Medicine The University of Tokyo Tokyo Japan; ^2^ Department of Clinical Epidemiology and Health Economics, School of Public Health The University of Tokyo Tokyo Japan; ^3^ Department of Real‐World Evidence, Graduate School of Medicine The University of Tokyo Tokyo Japan; ^4^ Department of Obstetrics and Gynecology, Reproductive Medicine Chiba University Chiba Japan; ^5^ Department of Social Medicine National Center for Child Health and Development Tokyo Japan; ^6^ Department of Obstetrics and Gynecology Teikyo University School of Medicine Tokyo Japan; ^7^ Teikyo Academic Research Center Teikyo University Tokyo Japan

**Keywords:** coagulation tests, contraceptives, drug prescriptions, practice guidelines as topic, thromboembolism

## Abstract

**Objective:**

Combined oral contraceptives (COCs) for dysmenorrhea management have been covered by the Japanese universal health insurance system since 2008. Several public awareness events regarding COC prescriptions for dysmenorrhea were implemented between 2013 and 2015, including media reports on COC‐related fatalities in 2013, drug safety alerts in 2014, and new clinical guidelines in 2015. This study aimed to examine the trends in patient characteristics, coagulation testing, and thromboembolism incidence before and after these events.

**Methods:**

We used the JMDC claims database to identify patients with dysmenorrhea prescribed COCs between 2009 and 2021. We evaluated the annual trends in guideline‐listed thromboembolism risk factors (age ≥ 40 years, obesity, and smoking), frequency of patients undergoing coagulation testing among patients prescribed COCs, and thromboembolism incidence.

**Results:**

Of 860 840 patients with dysmenorrhea, 173 502 received COCs. The proportion of patients prescribed COCs decreased temporarily from 12.2% to 11.2% in 2014. The proportion of new COC prescriptions increased for women aged <40 years and decreased for those aged ≥40 years. The proportions of patients with obesity and smokers remained stable from 2016. The frequency of coagulation testing increased annually, rising sharply from 15% to 22% in 2014. The incidence of thromboembolism with treatment decreased from 11.8 to 9.9 per 10 000 person‐years from 2016 to 2021.

**Conclusion:**

Although no direct causal relationship is clear, public health awareness events might have influenced clinical practices, including the preferential selection of lower‐risk patients and promotion of coagulation screening, possibly resulting in a decline in the incidence of thromboembolism.

## 1 INTRODUCTION

Combined oral contraceptives (COCs) were first introduced in the 1950s for the purpose of contraception.^1^ Since then, additional benefits of COCs beyond contraception (e.g., amelioration of dysmenorrhea) have garnered recognition. In 2008, COCs received coverage by universal health insurance in Japan for the treatment of dysmenorrhea.^2^

However, COCs are also associated with an elevated risk of thromboembolic events, such as venous thromboembolism (VTE), including deep vein thrombosis (DVT) and pulmonary embolism (PE), and arterial thromboembolism (ATE).^3^ Previous studies have estimated that the incidence of VTE and ATE among COC users is 1.5 to 6 per 10 000 person‐years.^4^, ^5^, ^6^

In 2013, two fatal cases of thromboembolism linked to the use of 20 μg ethinyl estradiol/drospirenone (EE/DRSP) for dysmenorrhea were widely reported in the Japanese media. In response, in January 2014, the government instructed pharmaceutical manufacturers to distribute a “Blue Letter,” a drug safety alert intended to inform healthcare professionals.^7^ These events have heightened public concern regarding the safety of COCs. Subsequently, in November 2015, the Japan Society of Obstetrics and Gynecology and the Japan Society for Menopause and Women's Health issued new clinical guidelines to promote safer and better‐informed use of COCs.^8^ The details of these events are provided in Supplementary Table S1. These guidelines emphasized the importance of identifying high‐risk individuals before initiating COCs, including patients aged ≥40 years, those with a body mass index (BMI) ≥30 kg/m^2^, and smokers. For patients at a high risk of thromboembolism, the guidelines recommend performing coagulation testing. However, the guidelines state that routine coagulation testing for all COC users is unnecessary.

Previous studies have assessed the incidence of thromboembolic events before the publication of the 2015 guidelines. For example, in a 2014 survey of 143 gynecologists, 33% reported a decrease in COC prescriptions for dysmenorrhea following the 2013 media reports.^9^

However, it is unclear how the series of public awareness events, including the 2013 media reports, 2014 drug safety alert, and 2015 guidelines, influenced the prescription patterns of COCs for dysmenorrhea and the incidence of thromboembolic events. This study aimed to assess the impact of these events on COC use and thromboembolic events in Japan.

## 2 MATERIALS AND METHODS

### 2.1 Ethics statements

This study was approved by the Institutional Review Board of the University of Tokyo, and the requirement for informed consent was waived owing to the de‐identified nature of the data.

### 2.2 Data source

This retrospective cohort study used the JMDC database (JMDC, Tokyo, Japan) from January 2009 to December 2021. The database contains monthly health insurance claims and health check‐up records. As of 2022, the database included the de‐identified data of over 17 million individuals.^10^ The database is sourced from health insurance societies that cover employees of large companies in Japan and their dependents aged under 75 years.^11^

The JMDC database contains demographic characteristics, diagnoses, insurance claims (for outpatient visits, hospitalization, drugs, and procedures), and health check‐up results. The demographic data include de‐identified personal identification, birth month, sex, and insurance enrollment period. Diagnoses are recorded using the International Classification of Diseases, Tenth Revision (ICD‐10) codes. The prescription data are classified include drug names, dosage, prescription dates, prescribing institutions, and classifications according to the World Health Organization Anatomical Therapeutic Chemical system. Medical procedures are recorded based on original Japanese procedure codes. Health check‐up data include BMI, smoking status, and lifestyle‐related information.

The JMDC database does not include prescriptions for COCs used for contraceptive purposes because they are not covered by the universal health insurance system in Japan.

### 2.3 Study design and population

We identified patients with dysmenorrhea between January 2009 and December 2021 using the following ICD‐10 codes: adenomyosis and endometriosis (N80), uterine fibroids (D25), and dysmenorrhea (N94.4, N94.5, and N94.6). From this population, we identified patients who were prescribed COCs. COCs were defined as formulations approved in Japan for the treatment of dysmenorrhea, including 35 μg EE/norethisterone (NET), 20 μg EE/DRSP, 20 μg EE/NET, and 20 μg EE/levonorgestrel (LNG). Patients were excluded if they had been diagnosed with thromboembolism or had received treatment for thromboembolism 1 year before COC initiation. The definitions are provided in Table S2.

### 2.4 Outcomes

We identified age ≥40 years, BMI ≥30.0 kg/m^2^, and smoking status from among the conditions listed in the guidelines requiring cautious prescription of COCs. We evaluated (i) all coagulation tests and (ii) D‐dimer alone. Thromboembolism was evaluated as all thromboembolic events and separately as ATE and VTE. Initially, the thromboembolic events were assessed based solely on the ICD‐10 codes. VTE was further categorized into DVT, PE, and others. Subsequently, the outcomes were evaluated based on the diagnosis and treatment of thromboembolism (definitions are provided in Table S2).

### 2.5 Statistical analysis

We used the Cochran–Armitage test to analyze the annual trends in COC prescription. In addition to the overall trends during the study period, we conducted a trend analysis for the period spanning 2016 to 2021 to assess the changes after the abovementioned events.

First, we examined the annual trends in the proportion of patients with dysmenorrhea who were prescribed COCs. To evaluate the changes in hormonal therapies other than COCs, we similarly assessed dienogest, gonadotropin‐releasing hormone analogs, and LNG‐releasing intrauterine systems (IUSs) (definitions are provided in Table S2). Second, we analyzed annual trends in the characteristics of patients who were newly prescribed COCs for dysmenorrhea. The patient characteristics included age group (<20, 21–29, 30–39, and ≥ 40 years), BMI (<18.5, 18.5–24.9, 25.0–29.9, ≥ 30.0 kg/m^2^, and missing data), and smoking status (smoker, non‐smoker, and missing data). The proportion was calculated as the number of patients newly prescribed COCs in each category divided by the total number of patients with dysmenorrhea in the same category. Third, we examined the annual trends in the distribution of each COC formulation. Fourth, we analyzed the annual trends in the frequency of patients prescribed COCs who underwent coagulation testing. Finally, we evaluated the annual trends in the incidence of thromboembolism per 10 000 person‐years among patients prescribed COCs. We also calculated the annual average number and annual average incidence of thromboembolic events per 10 000 person‐years for three periods: before (2009–2012), during (2013–2015), and after (2016–2021) the public awareness events.

A two‐sided significance level of *P* < 0.05 was set for all tests. All statistical analyses were performed using Stata 18, except for trend analyses, which were conducted using R 4.3.1.

## 3 RESULTS

**FIGURE 1:**
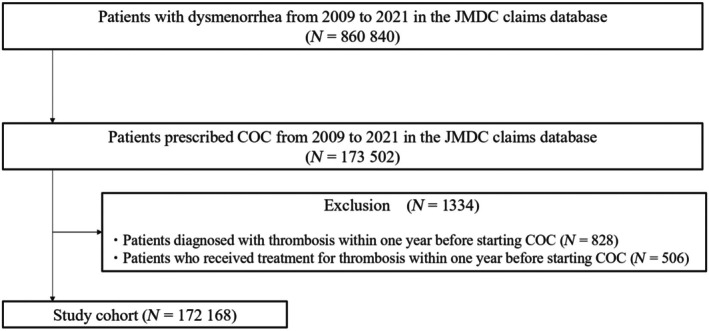
Patient selection flow chart. Patients with dysmenorrhea were identified using the International Classification of Diseases, Tenth Revision (ICD‐10) codes. COC, combined oral contraceptive.

Of 860 840 patients with dysmenorrhea, 173 502 were prescribed COCs, with 172 168 included in the study after exclusions (Figure 1).

**FIGURE 2:**
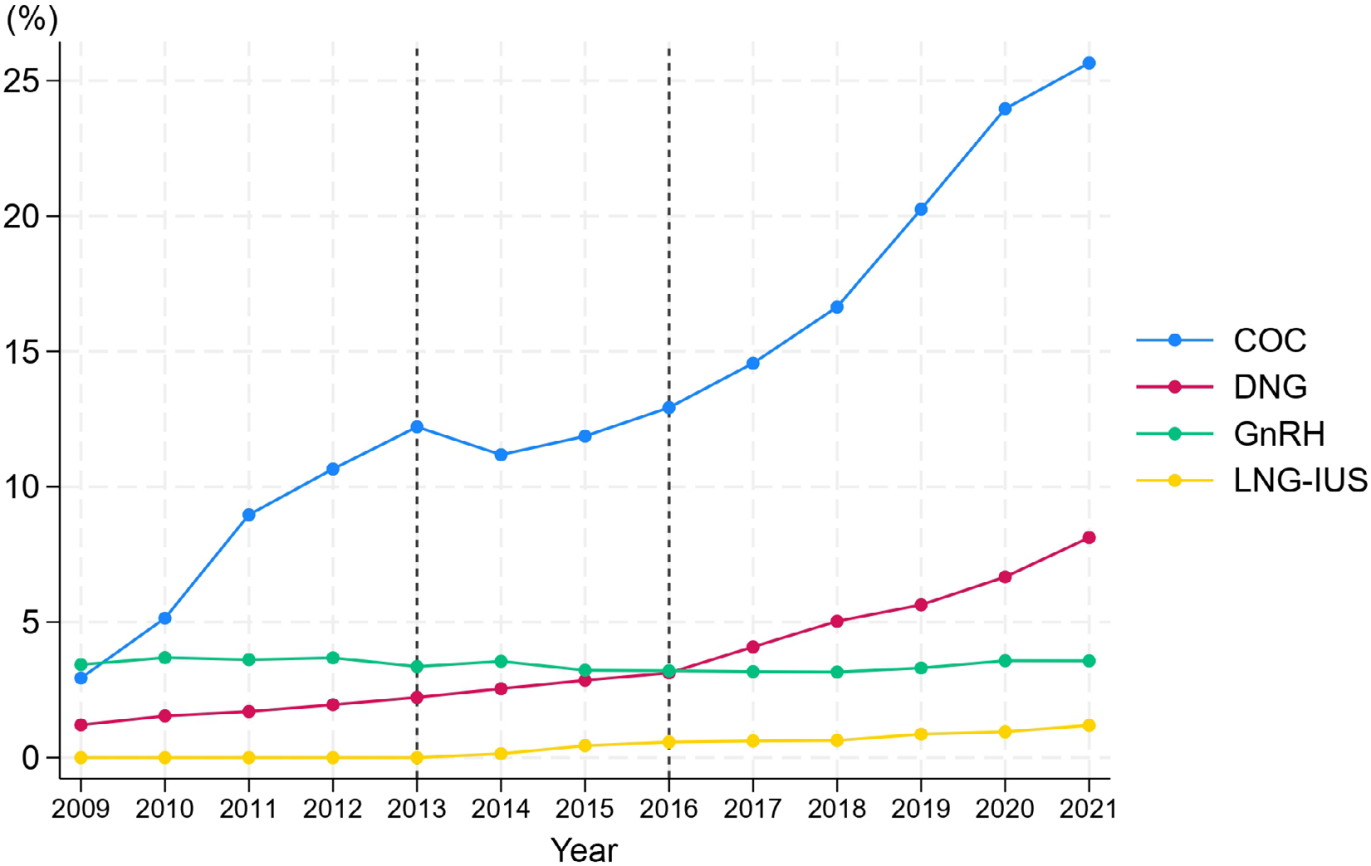
Annual trends in the proportion of patients with dysmenorrhea who were prescribed hormonal therapy between 2009 and 2021. Patients with dysmenorrhea were identified using the International Classification of Diseases, Tenth Revision codes. The dashed vertical lines denote the 2013–2015 period, during which the public awareness events occurred. COC, combined oral contraceptive; DNG, dienogest; GnRH, gonadotropin‐releasing hormone analog; LNG‐IUS, levonorgestrel‐releasing intrauterine system.

The proportion of patients prescribed COCs increased from 2.9% in 2009 to 25.7% in 2021. However, after 2014, this proportion temporarily decreased from 12.2% to 11.2%. The proportion of patients prescribed dienogest and LNG‐IUS increased from 2.5% and 0.1% in 2014 to 8.1% and 1.2% in 2021, respectively (Figure 2).

**FIGURE 3:**
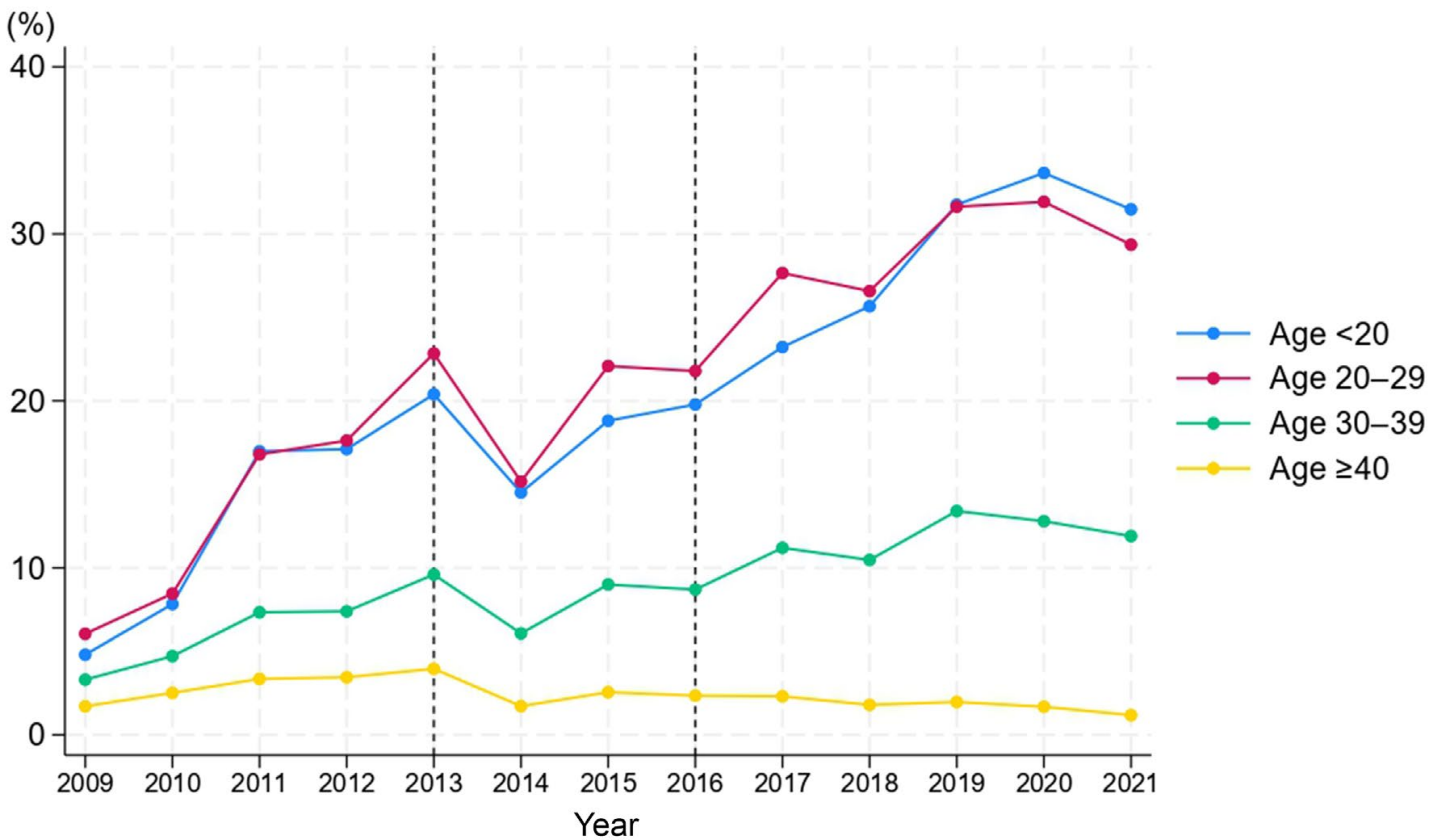
Annual trends in patients with dysmenorrhea who were newly prescribed combined oral contraceptives stratified by age. The dashed vertical lines denote the 2013–2015 period, during which the public awareness events occurred. Patients with dysmenorrhea were identified using the International Classification of Diseases, Tenth Revision codes. The proportions were calculated as the number of patients with dysmenorrhea who were newly prescribed combined oral contraceptives divided by the total number of patients with dysmenorrhea in each age category. *P* for trend (2016–2021) by age group: <20 years, *P* < 0.001; 20–29 years, *P* < 0.001; 30–39 years, *P* < 0.001; ≥40 years, *P* < 0.001.

**FIGURE 4:**
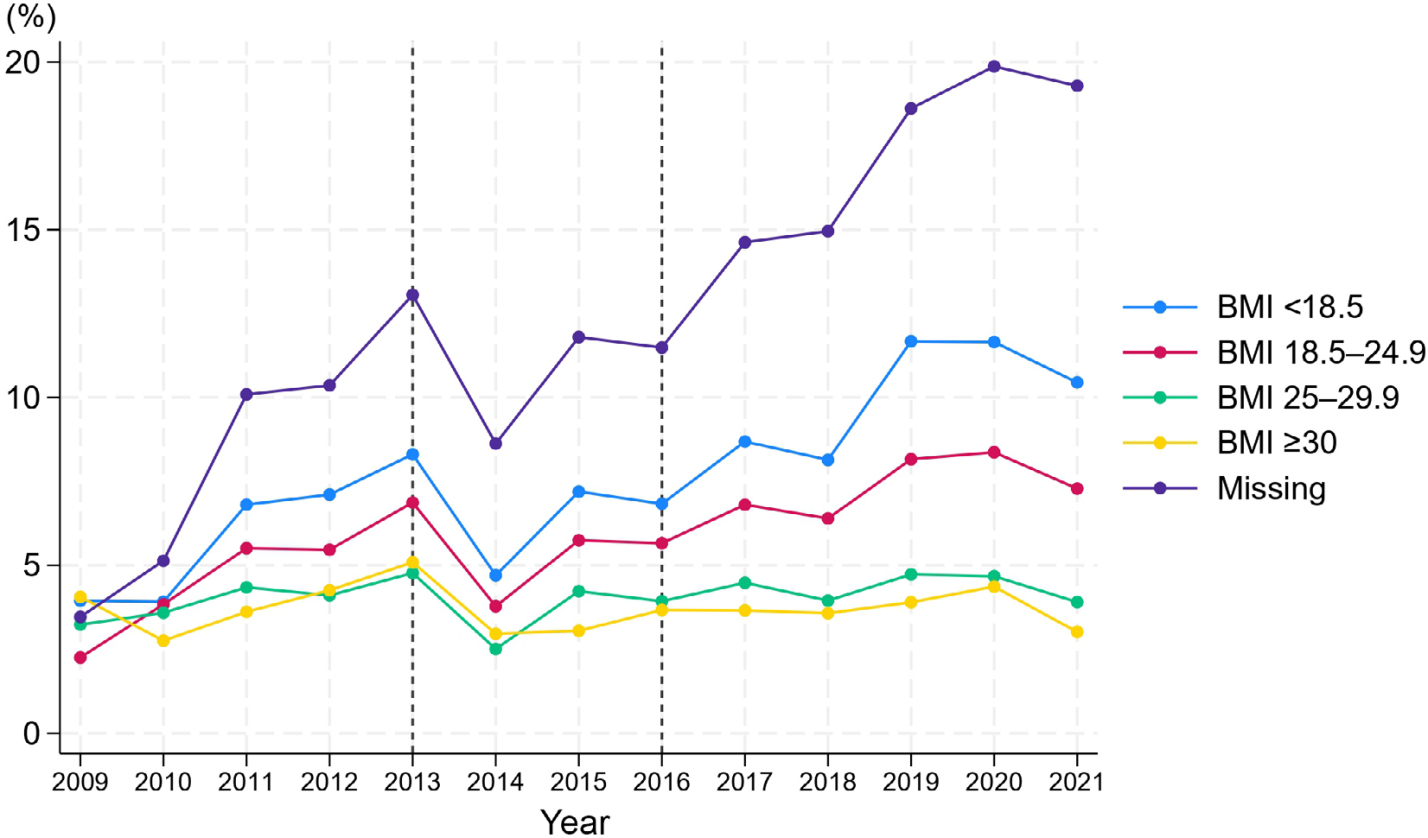
Annual trends in patients with dysmenorrhea who were newly prescribed combined oral contraceptives stratified by body mass index. The dashed vertical lines denote the 2013–2015 period, during which the public awareness events occurred. Patients with dysmenorrhea were identified using the International Classification of Diseases, Tenth Revision codes. BMI, body mass index. The proportions were calculated as the number of patients newly prescribed combined oral contraceptives divided by the total number of patients with dysmenorrhea in each BMI category. *P* for trend (2016–2021) by BMI group: <18.5, *P* < 0.001; 18.5–24.9, *P* < 0.001; 25.0–29.9, *P* = 0.962; ≥30.0, *P* = 0.335; missing, *P* < 0.001.

**FIGURE 5:**
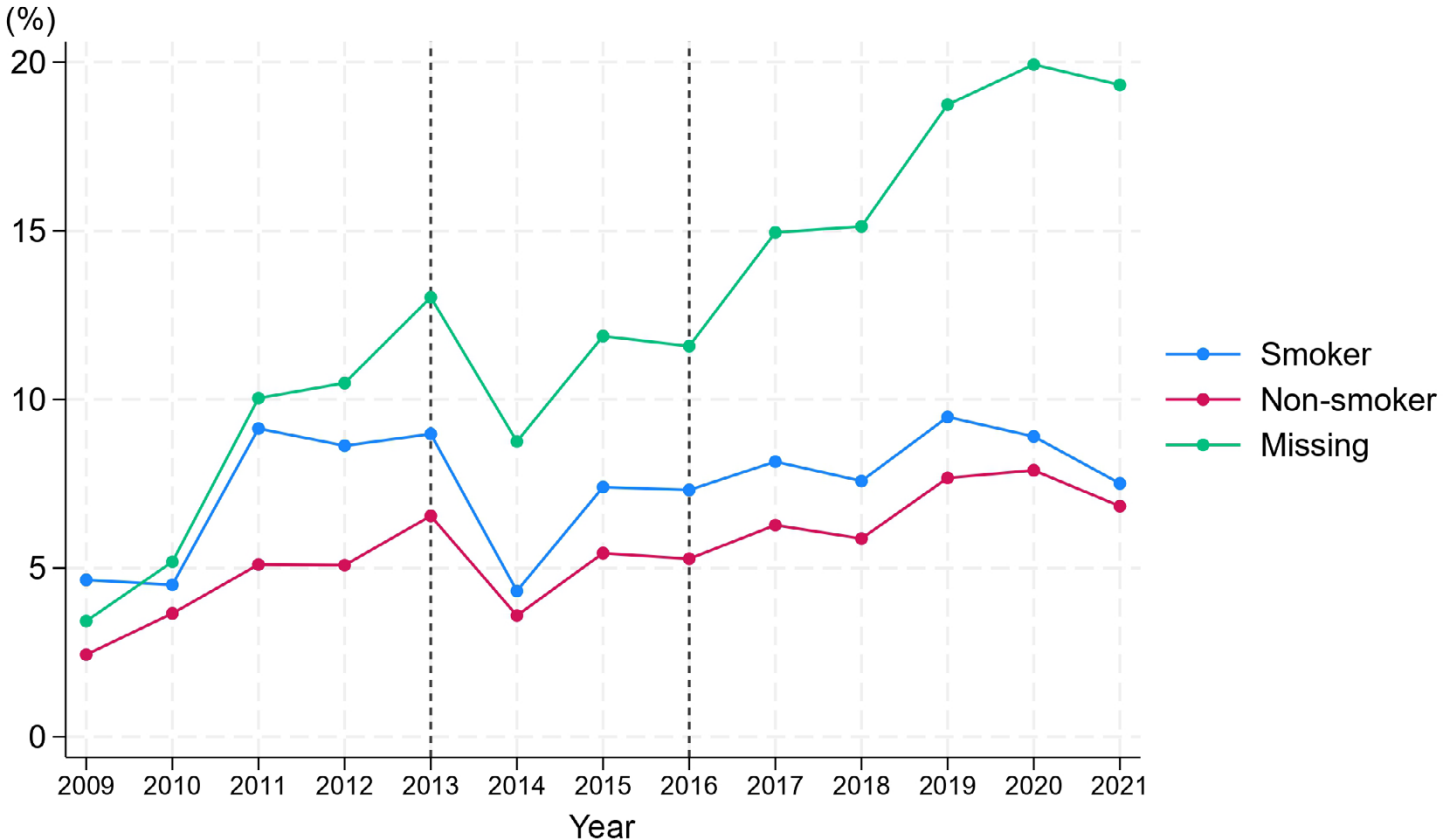
Annual trends in patients with dysmenorrhea who were newly prescribed combined oral contraceptives based on smoking status. The dashed vertical lines denote the 2013–2015 period, during which the public awareness events occurred. Patients with dysmenorrhea were identified using the International Classification of Diseases, Tenth Revision codes. The proportions were calculated as the number of patients newly prescribed combined oral contraceptives divided by the total number of patients with dysmenorrhea in each smoking status category. *P* for trend (2016–2021) by smoking status: Smoker, *P* = 0.175; non‐smoker, <0.001; missing, *P* < 0.001.

Figures 3, 4, 5 show the annual trends in patients who were newly prescribed COCs for dysmenorrhea, stratified by age, BMI, and smoking status. After 2016, the proportion of patients aged <40 years increased, while that of patients aged ≥40 years decreased (*P* < 0.001). The proportion of patients with BMI <25.0 kg/m^2^ increased significantly (*P* < 0.001), whereas no significant change was observed in the proportions of patients with BMI 25.0–29.9 kg/m^2^ and BMI ≥30.0 kg/m^2^ after 2016 (*P* = 0.962 and *P* = 0.335, respectively). Although the proportion of smokers did not change significantly after 2016 (*P* = 0.175), that of non‐smokers increased significantly (*P* < 0.001).

**FIGURE 6:**
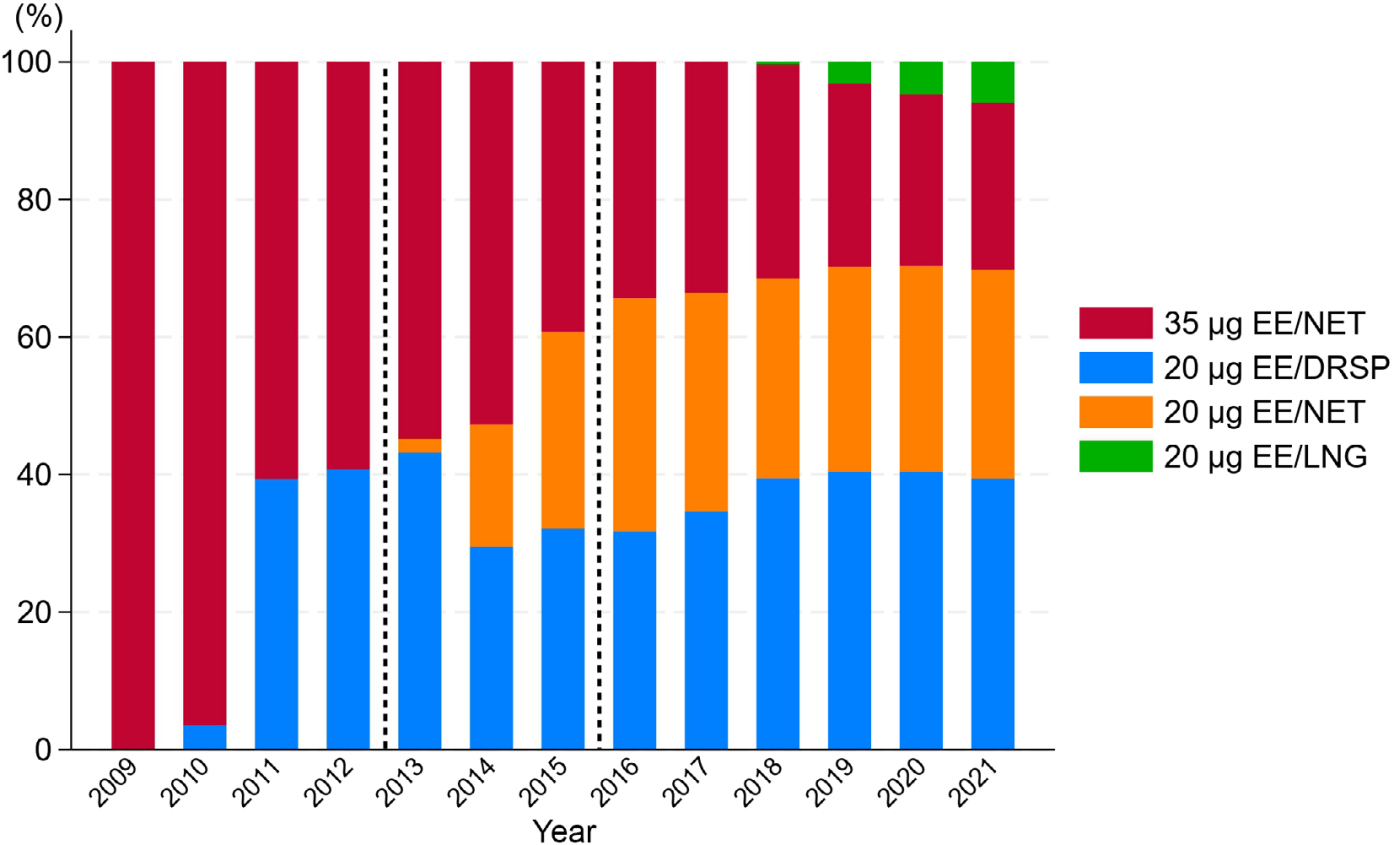
Annual trends in formulation types among combined oral contraceptive users between 2009 and 2021. The dashed vertical lines denote the 2013–2015 period, during which the public awareness events occurred. DRSP, drospirenone; EE, ethinyl estradiol; LNG, levonorgestrel; NET, norethisterone.

The proportion of 20 μg EE/DRSP decreased from 43% to 30% in 2014, while that of 20 μg EE/NET increased from 2% to 18% (Figure 6).

**FIGURE 7:**
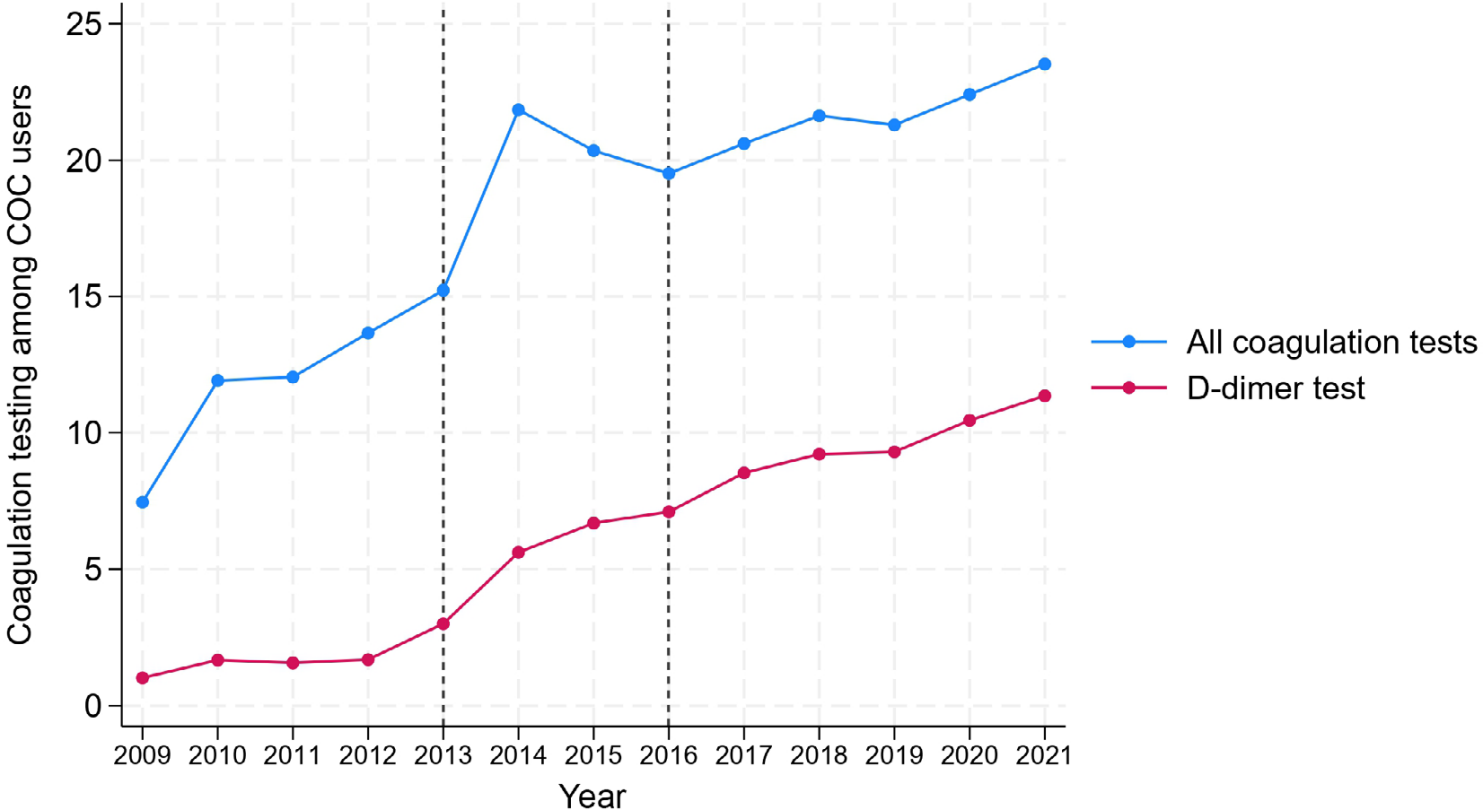
Annual trends in the frequency of combined oral contraceptive users who underwent coagulation testing between 2009 and 2021. The dashed vertical lines denote the 2013–2015 period, during which the public awareness events occurred. “All coagulation tests” refers to the frequency of patients who underwent at least one coagulation test, including prothrombin time, activated partial thromboplastin time, fibrinogen, D‐dimer, and others (Table S2). The “D‐dimer test” refers specifically to the proportion of patients who underwent a D‐dimer test.

The proportion of all coagulation tests, specifically the D‐dimer test alone, increased from 2009 to 2021. For all coagulation tests, a sharp temporary increase from 15% to 22% was evident in 2014 (Figure 7).

**FIGURE 8:**
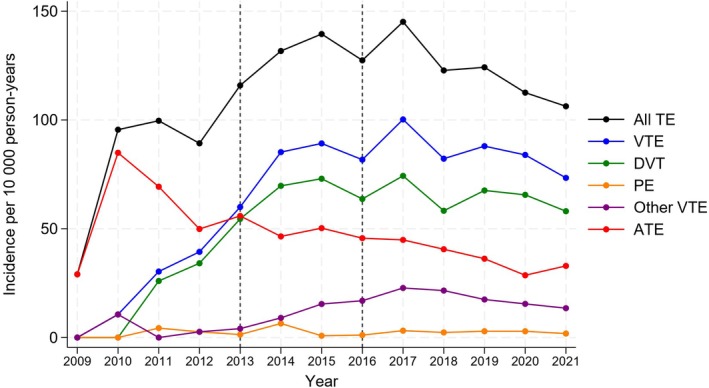
Annual incidence of thromboembolism diagnosis per 10 000 combined oral contraceptive users between 2009 and 2021. The dashed vertical lines denote the 2013–2015 period, during which the public awareness events occurred. All TEs consisted of VTE and ATE, with VTE further divided into DVT, PE, and other VTEs. ATE, arterial thromboembolism; COC, combined oral contraceptive; DVT, deep vein thrombosis; PE, pulmonary embolism; TE, thromboembolic events; VTE, venous thromboembolism.

**FIGURE 9:**
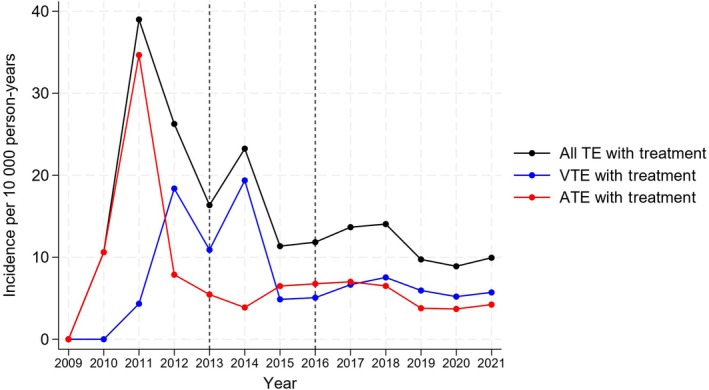
Annual incidence of thromboembolism receiving treatment per 10 000 combined oral contraceptive users between 2009 and 2021. The dashed vertical lines denote the 2013–2015 period, during which the public awareness events occurred. All TEs with treatment included VTE with treatment and ATE with treatment. ATE, arterial thromboembolism; COC, combined oral contraceptive; TE, thromboembolic event; VTE, venous thromboembolism.

Table 1 presents the annual trends in the incidence of thromboembolic events. None of the thromboembolic events showed a significant upward trend after 2016 (Figure 8). After 2016, the incidence of all thromboembolic events decreased from 127.3 to 105.4 per 10 000 person‐years. Similarly, the analysis of thromboembolic events requiring treatment revealed no significant upward trend. After 2016, the incidence of all thromboembolic events requiring treatment decreased from 11.8 to 9.9 per 10 000 person‐years (Figure 9). Tables S3 and S4 present the annual average number and incidence of thromboembolic events before, during, and after 2013–2015. The annual average incidence of all thromboembolic events was 131.1 per 10 000 person‐years during 2013–2015, which decreased to 117.6 during 2016–2021.

**TABLE 1:** Annual trends in the incidence of thromboembolism per 10 000 person‐years prescribed combined oral contraceptives between 2009 and 2021.

	2009	2010	2011	2012	2013	2014	2015	2016	2017	2018	2019	2020	2021	2009–2021 p for trend	2016–2021 p for trend
All thromboembolic events	33.9	98.9	100.7	89.9	116.2	131.9	139.5	127.3	144.9	122.5	123.9	112.0	105.4	< 0.001	< 0.001
VTE	0.0	11.0	30.6	39.6	60.1	85.3	89.2	81.7	100.1	82.0	87.7	83.5	72.7	0.538	< 0.001
Deep vein thrombosis	0.0	0.0	26.3	34.4	54.7	69.8	73.0	63.7	74.2	58.1	67.4	65.3	57.5	0.681	0.049
Pulmonary embolism	0.0	0.0	4.4	2.6	1.4	6.5	0.8	1.1	3.2	2.3	2.9	2.9	1.8	0.672	0.735
Other VTE	0.0	11.0	0.0	2.6	4.1	9.1	15.4	16.9	22.8	21.5	17.4	15.4	13.4	0.463	< 0.001
ATE	33.9	87.9	70.1	50.2	56.0	46.5	50.3	45.6	44.8	40.5	36.1	28.5	32.6	< 0.001	< 0.001
All thromboembolic events with treatment	0.0	11.0	39.4	26.4	16.4	23.3	11.4	11.8	13.7	14.0	9.7	8.9	9.9	< 0.001	0.021
VTE with treatment	0.0	0.0	4.4	18.5	10.9	19.4	4.9	5.1	6.7	7.5	5.9	5.2	5.7	0.002	0.446
ATE with treatment	0.0	11.0	35.0	7.9	5.5	3.9	6.5	6.8	7.0	6.5	3.8	3.7	4.2	< 0.001	0.009

Abbreviations: ATE, arterial thromboembolism; VTE, venous thromboembolism.

## 4 DISCUSSION

The present study examined the trends in COC prescriptions in Japan after a series of public awareness events, including the 2013 media reports on COC‐related fatal cases, 2014 drug safety alert, and 2015 new clinical guidelines, using data extracted from a large‐scale claims database. After these events, the proportion of patients who were newly prescribed COCs increased among those aged <40 years and decreased among those aged ≥40 years. The proportion of patients with a BMI >25.0 kg/m^2^ or those who smoked remained stable after these events. The proportion of patients who received prescriptions and underwent coagulation testing increased annually, with a temporary sharp rise in 2014. The incidence of thromboembolic events showed a declining trend after these events.

When stratified by age, new COC prescriptions increased among patients aged <40 years and declined among patients aged ≥40 years after 2016. One possible explanation is the influence of the 2013–2015 events, which might have led gynecologists to avoid prescribing COCs to patients aged ≥40 years who are categorized as requiring cautious use in the guidelines. In fact, a survey reported that gynecologists were less likely to prescribe COCs to women aged ≥40 years after the 2013 media reports.^9^ Additionally, the expansion of alternative treatment options in Japan might have contributed to this trend. For example, LNG‐IUS received insurance coverage for dysmenorrhea in 2014.^12^ Further, generic formulations of dienogest became available in 2017.^13^ These drugs pose a lower risk of thromboembolic events than COCs.^14^, ^15^, ^16^, ^17^ In this study, the proportion of patients prescribed dienogest and LNG‐IUS exhibited an uptick during the study period.

From 2016 to 2021, the proportion of new COC prescriptions increased among patients with a BMI <25.0 kg/m^2^, whereas no significant change was apparent among those with a BMI ≥25.0 kg/m^2^. One possible explanation is that gynecologists might have been reluctant to prescribe COCs to patients with a high BMI after the events. In Japan, a BMI ≥25.0 kg/m^2^ is classified as obesity, whereas the World Health Organization defines obesity as a BMI ≥30.0 kg/m^2^.^18^ Although the 2015 guidelines recommended cautious prescription of COCs only for patients with a BMI >30.0 kg/m^8^, gynecologists might have also avoided prescribing COCs to those with a BMI ≥25.0 kg/m^2^. Indeed, a 2014 survey of Japanese gynecologists reported that 18.5% had adopted a lower BMI threshold for COC prescriptions.^9^ Moreover, a Japanese study showed that women with a BMI ≥25.0 kg/m^2^ faced an elevated risk of thromboembolism.^19^ Based on this finding, gynecologists might have been less likely to prescribe COCs not only to patients with a BMI ≥30.0 kg/m^2^ but also to those with a BMI ≥25.0 kg/m^2^.

While the proportion of new COC prescriptions increased among non‐smokers, there was no significant change among smokers following the events. One possible explanation is that gynecologists might have been reluctant to prescribe COCs to smokers after these events. The 2015 guidelines recommend cautious prescription of COCs to smokers.^8^ A 2014 survey of Japanese gynecologists reported that 27% had stopped prescribing COCs to smokers.^9^

There was a decline in prescriptions of 20 μg EE/DRSP in 2014, which might have been influenced by media reports in 2013 regarding fatal cases associated with 20 μg EE/DRSP. A previous survey reported that gynecologists tended to change COC formulations in response to media reports.^9^ Additionally, the launch of 20 μg EE/NET in September 2013 might have contributed to changes in prescription patterns.^20^ Previous studies suggested that lower doses of EE lowered the risk of thromboembolic events and that NET was associated with a lower thromboembolic risk than DRSP.^14^, ^21^, ^22^ These factors might have influenced both gynecologists and patients to prefer 20 μg EE/NET over 20 μg EE/DRSP.

The proportion of coagulation testing showed an overall increasing trend between 2009 and 2021. Notably, a sharp temporary increase in 2014 probably reflects enhanced screening following the media reports. A previous survey showed that some gynecologists increased coagulation screening after the 2013 media report.^9^ However, the 2015 guidelines indicated that D‐dimer testing possesses limited utility in predicting VTE and do not recommend routine coagulation testing.^8^ This suggests that coagulation testing is conducted more frequently than is clinically necessary.

The incidence of all thromboembolic events showed a declining trend following the abovementioned events. The events might have promoted more cautious COC use by both gynecologists and patients. For example, the guidelines recommended performing coagulation testing before initiating COCs and repeating the test after 6 months in patients at risk of thromboembolism; our study also showed an increase in the proportion of coagulation testing. Heightened awareness might also have been influenced by the 2015 guidelines, such as avoiding prolonged immobilization and paying closer attention to thromboembolism symptoms. Gynecologists might have refrained from prescribing COCs to high‐risk patients such as women aged ≥40 years. Further, the more widespread prescription of low‐dose estrogen formulations after 2013 might have contributed to the reduced thromboembolic risk.

In this study, the proportion of COC prescriptions (especially 20 μg EE/DRSP) temporarily declined in 2014. This suggests that media reports and drug safety alerts might have influenced decision‐making regarding COC prescriptions. A similar phenomenon was observed in the UK in 1995. Exaggerated media reports and strongly assertive safety alerts regarding the thromboembolic risks of COCs for contraception led to a marked reduction in prescriptions as well as increases in conception and abortion rates.^23^, ^24^ These observations indicate that media reports and safety alerts can significantly affect clinical practice; thus, accurate and balanced risk reports and alerts are important to avoid unintended adverse effects on patient care. In our study, the incidence of thromboembolism declined after the 2013–2015 public awareness events. Thus, based on our findings, there is no clear need to increase or decrease the intensity of public awareness activities at present. However, if the safety profile or usage patterns of COCs were to change in the future, the level of public awareness activities should be re‐evaluated accordingly. This study had some limitations. First, it was not possible to confirm the patients' actual adherence to the prescribed COCs. Additionally, because COC prescriptions for contraception were not captured in this database, the impact of the events on this indication of COCs could not be evaluated. Further, it is possible that some women who were using COCs for contraception presented to clinicians with dysmenorrhea instead, to receive insurance coverage. Second, important clinical details, such as the number of cigarettes smoked and pregnancy status, were not available in the claims data. Third, misclassification of diagnosis might have occurred. However, we used both diagnostic and treatment records to improve the accuracy of thrombotic event identification. Fourth, although this study primarily evaluated the impact of the abovementioned events, other societal or regulatory events, as well as the rise of online media, might also have influenced prescription patterns. Fifth, because the JMDC claims database consists of employees of large companies and their dependents, the findings might not be generalizable to the entire Japanese population.

## 5 CONCLUSION

In conclusion, this study provided a comprehensive assessment of nationwide trends in COC prescriptions for dysmenorrhea before and after public awareness events from 2013 to 2015. Prescriptions declined notably among women aged ≥40 years after the events. Prescriptions of lower‐dose estrogen formulations increased, and coagulation testing became more frequent. Although the direct effect of the events is unclear, the incidence of overall thromboembolic events decreased after 2016.

## AUTHOR CONTRIBUTIONS

All authors participated in the study design and data interpretation. HM and HY managed the databases. RI and YS analyzed the data and performed statistical analyses. RI drafted the first version of the manuscript. YS, KK, YO, and HY contributed to the final version of the manuscript. The manuscript was finalized and approved by all the co‐authors.

## FUNDING INFORMATION

This work was supported by grants from the Ministry of Health, Labour and Welfare, Japan (grant number: 23AA2003), and the Japan Agency for Medical Research and Development (grant number: 25gk0210042h0001).

## CONFLICT OF INTEREST STATEMENT

The authors have no conflicts of interest to declare.

## Supporting information


Data S1.

## Data Availability

The data supporting the findings of this study are available from JMDC. Restrictions apply to the availability of these data, which were used under license for this study. Data can be accessed with the permission of JMDC. Requests to access these datasets should be directed to https://www.jmdc.co.jp/en/inquiry.
