# Supplementary material for: Prescription trends for combined oral contraceptives and thromboembolism incidence in Japan before and after public awareness events in 2013–2015
Source: Int J Gynaecol Obstet. 2026 Jan 26;174(1):299–308. doi: 10.1002/ijgo.70835 (PMC13278627; doi:10.1002/ijgo.70835)
Supplement: Supplementary file 1 — Data S1. [file IJGO-174-299-s001.docx]

Supplementary Table S1. Details of the public awareness events between 2013 and 2015

| August 2013 | The first fatal case related to ethinyl estradiol/drospirenone was reported in the Japanese media^1^. |
| --- | --- |
| October 2013 | The second fatal case related to ethinyl estradiol/drospirenone was reported in the Japanese media^1^. |
| December 2013 | At a press conference, the government reported a total of 11 deaths attributed to the adverse effects of combined oral contraceptives between 2004 and 2013^2^. |
| December 2013 | The Japan Society of Obstetrics and Gynecology released a public statement for women titled “*Teiyoryo piru no fukusayo ni tsuite shinpai shite orareru josei e”* [To women concerned about the adverse effects of combined oral contraceptives] (in Japanese)^2^. |
| January 2014 | Following the report of the third fatal case potentially associated with ethinyl estradiol/drospirenone, the government instructed pharmaceutical manufacturers to issue a “Blue Letter,” i.e., a drug safety alert for healthcare professionals^1^. |
| November 2015 | The Japan Society of Obstetrics and Gynecology published new clinical guidelines to promote safer and better-informed use of combined oral contraceptives, in response to growing public concerns about the safety of combined oral contraceptives. |

References

1 Ministry of Health, Labour and Welfare. Gekkei konnan-sho chiryozai “Yazu haigojo” toyo kanja de no kessansho ni kansuru chuikanki [Safety alert regarding thrombosis in patients administered the dysmenorrhea treatment “Yaz combination tablet”] (in Japanese). http://www.mhlw.go.jp/stf/houdou/0000034892.html. Accessed December 3, 2025.

2 Kitamura K. OC to kessensho [Oral contraceptives and thrombosis] (in Japanese). *Clinical Gynecology and Obstetrics*. 2014; 68(7): 714–720.

Supplementary Table S2. Definitions of the Inclusion and Exclusion Criteria and Outcomes

|  | Definition |
| --- | --- |
| Combined oral contraceptive | 35 μg ethinylestradiol/norethisterone (WHO-ATC code: G03FA01 and Japanese brand names)  20 μg ethinylestradiol/drospirenone (G03FA17)  20 μg ethinylestradiol/norethisterone (G03FA01 and Japanese brand names)  20 μg ethinylestradiol/levonorgestrel (G03FA11) |
| Dienogest | Dienogest (WHO-ATC code: G03DB08) |
| Levonorgestrel-releasing intrauterine system | Levonorgestrel (WHO-ATC code: G02BA03) |
| Gonadotropin-releasing hormone analog | Leuprorelin (WHO-ATC code: L02AE01)  Buserelin (WHO-ATC code: L02AE02)  Relugolix (WHO-ATC code: L02BX04) |
| Thromboembolism diagnoses | All thromboembolic events were defined as the sum of VTE and ATE.  VTE  Deep vein thrombosis (ICD-10: I80); Pulmonary embolism (I26); Other venous embolism and thrombosis (I82)  ATE  Ischemic heart diseases (I20–25); Arterial embolism and thrombosis (I74); Cerebral infarction (I63); Transient cerebral ischemic attack, unspecified (G45.9) |
| Treatment for thromboembolism | Medication  Anticoagulant therapy and thrombolytic therapy (WHO-ATC code: B01)  Procedures (Japanese medical procedural codes):  Cerebral thrombectomy (K164-3)  Endovascular neurosurgery (K178)  Sigmoid sinus thrombosis surgery (K316)  Atrial thrombectomy (K543)  Percutaneous coronary angioplasty (K546)  Percutaneous coronary atherectomy (K547)  Percutaneous coronary angioplasty using specialized catheters (K548)  Percutaneous coronary stent placement (K549)  Intracoronary thrombolytic therapy (K550)  Coronary endarterectomy (K551)  Coronary artery or aortic bypass graft surgery (K552)  Pulmonary endarterectomy (K592-2)  Pulmonary vein thrombectomy (K593)  Arterial embolus removal (K608)  Arterial endarterectomy (K609)  Vascular dilation or thrombectomy of limbs (K616)  Venous thrombectomy (K619)  Inferior vena cava filter placement (K620) |
| Coagulation test | All coagulation tests  Prothrombin Time, Activated Partial Thromboplastin Time, D-dimer, Fibrinogen, Cryofibrinogen, Antithrombin, Fibrin/Fibrinogen Degradation Products, Fibrin Monomer Complex, Protein S, Protein C, Thrombin-Antithrombin Complex  D-dimer |

VTE, venous thromboembolism; ATE, arterial thromboembolism; WHO-ATC, World Health Organization Anatomical Therapeutic Chemical; ICD-10, International Classification of Diseases, Tenth Revision

Supplementary Table S3. Annual average number of thromboembolisms before, during, and after 2013–2015

|  | 2009–2012 | 2013–2015 | 2016–2021 |
| --- | --- | --- | --- |
| All thromboembolic events | 16.8 | 119.7 | 592.2 |
| VTE | 4.8 | 61.3 | 318.5 |
| Deep vein thrombosis | 0.5 | 2.3 | 12.2 |
| Pulmonary embolism | 0.5 | 9.7 | 84.3 |
| Other VTE | 11.0 | 46.3 | 177.2 |
| ATE | 5.0 | 14.7 | 53.3 |
| All thromboembolic events with treatment | 3.0 | 5.0 | 23.7 |
| VTE with treatment | 5.8 | 73.3 | 415.0 |
| ATE with treatment | 2.0 | 9.7 | 29.7 |

VTE, venous thromboembolism; ATE, arterial thromboembolism

Supplementary Table S4. Annual average incidence of thromboembolism across before, during, and after 2013–2015 per 10,000 person-years

|  | 2009–2012 | 2013–2015 | 2016–2021 |
| --- | --- | --- | --- |
| All thromboembolic events | 92.1 | 131.1 | 117.6 |
| VTE | 26.1 | 67.2 | 63.3 |
| Deep vein thrombosis | 2.7 | 2.6 | 2.4 |
| Pulmonary embolism | 2.7 | 10.6 | 16.7 |
| Other VTE | 60.5 | 50.8 | 35.2 |
| ATE | 27.5 | 16.1 | 10.6 |
| All thromboembolic events with treatment | 16.5 | 5.5 | 4.7 |
| VTE with treatment | 31.6 | 80.3 | 82.4 |
| ATE with treatment | 11.0 | 10.6 | 5.9 |

VTE, venous thromboembolism; ATE, arterial thromboembolism
